# Point Cloud Geometry Compression Based on Multi-Layer Residual Structure

**DOI:** 10.3390/e24111677

**Published:** 2022-11-17

**Authors:** Jiawen Yu, Jin Wang, Longhua Sun, Mu-En Wu, Qing Zhu

**Affiliations:** 1 Faculty of Information Technology, Beijing University of Technology, Beijing 100124, China; 2 Department of Information and Finance Managment, National Taipei University of Technology, Taipei 10608, Taiwan

**Keywords:** point cloud geometry compression, multi-layer residual module, progressive sampling

## Abstract

Point cloud data are extensively used in various applications, such as autonomous driving and augmented reality since it can provide both detailed and realistic depictions of 3D scenes or objects. Meanwhile, 3D point clouds generally occupy a large amount of storage space that is a big burden for efficient communication. However, it is difficult to efficiently compress such sparse, disordered, non-uniform and high dimensional data. Therefore, this work proposes a novel deep-learning framework for point cloud geometric compression based on an autoencoder architecture. Specifically, a multi-layer residual module is designed on a sparse convolution-based autoencoders that progressively down-samples the input point clouds and reconstructs the point clouds in a hierarchically way. It effectively constrains the accuracy of the sampling process at the encoder side, which significantly preserves the feature information with a decrease in the data volume. Compared with the state-of-the-art geometry-based point cloud compression (G-PCC) schemes, our approach obtains more than 70–90% BD-Rate gain on an object point cloud dataset and achieves a better point cloud reconstruction quality. Additionally, compared to the state-of-the-art PCGCv2, we achieve an average gain of about 10% in BD-Rate.

## 1. Introduction

Owing to a rapid innovation of visual capture technology, point clouds have been regarded as vital data to describe both 3D objects and scenes [1]. Therefore, point clouds have a broad range of applications in areas, such as autonomous driving and augmented reality. Point clouds collections are points in space, including the coordinates of geometry information. In addition, every point can have attribute information attached, including colors, normals and reflectances. Since point cloud data are usually very large in quantity and is disordered and irregular [2] in structure, when compared with image and video compression [3,4,5,6,7], how to efficiently compress point cloud data becomes a challenging task.

Point cloud compression can be divided into geometric compression and attribute compression according to the compression object. The geometric information is coded independently, and the attribute information needs to be coded based on the known geometric structure information. The irregular spatial distribution of point clouds makes it difficult to compress. Therefore, it is necessary to convert point clouds into normalized and organized data structures, such as volume model, tree structure, grid model and a multi-view image. Hence using the traditional methods, the encoding techniques based on tree structure [8,9,10,11,12,13], surface approximation [13,14] and mapping [15,16,17,18,19], are several representative encoding schemes of point cloud geometry. The existing standard compression methods for point clouds are released by MPEG [1] consisting of geometry-based PCC (G-PCC) applied to static point clouds and video-based PCC (V-PCC) applied to dynamic point clouds, which are both typical methods. Moreover, with the development of artificial intelligence technology, recent works [20,21,22,23] have applied deep learning to Point Cloud Compression (PCC), e.g., PointNet [24], which greatly improves the compression performance of point clouds, including point-based point cloud compression methods and voxel-based compression methods.

In this work, we propose a multi-layer residual architecture for Point Cloud Geometry Compression (PCGC) based on voxel using sparse convolution [25]. The main contributions of this work are as follows:

- A multi-layer residual module is introduced to take advantage of the distortion in entropy coding by geometric subtraction, to constrain the accuracy of the sampling process at the encoder side;
- We employ sparse convolution to design a multi-layer residual module and progressive up-sampling reconstruction for efficient processing of sparse tensors at low complexity;
- We adopt a novel joint loss distortion by designing a multi-layer residual loss obtained by multi-layer residual operation to improve the quality of the reconstructed point cloud.

Extensive experimental results demonstrate that our method can lessen the feature information loss caused during the quantization process and improve the accuracy of point cloud information extraction. Our method also performs well in subjective visual quality. In addition, our method outperforms G-PCC [1] and V-PCC [1] substantially at BD-Rate gain and achieves a 10% BD-Rate gain over the state-of-the-art method PCGCv2 [20].

The remainder of this paper is as follows: Section 2 provides a summary overview of the related work; Section 3 describes our proposed multi-layer residual architecture; Section 4 gives the experimental details and presents the experimental results; The Section 5 briefly concludes the paper.

## 2. Related Work

### 2.1. Conventional PCGC

Conventional non-deep learning based PCC methods include MPEG G-PCC [1], V-PCC [1], etc. According to the data structure into which the point cloud is converted, it can be divided into tree structure based, surface approximation based and mapping based encoding methods.

#### 2.1.1. Tree Structure Based PCGC

The encoding method based on tree structure is suitable for sparse point cloud, which is simple, direct and effective, and was the earliest to be developed. Many works store point cloud data in such octrees and use specific entropy models, such as adaptive histogram, parent-child node context [8], estimation based on plane approximation [9] or nearest neighbor [10]. To remove temporal redundancy in point cloud sequences, Kammerl et al. [11] encoded the differences between consecutive octrees, while Mekuria et al. [12] use encoded rigid-body transformed blocks, with both methods using empirical histograms for range coding when coding. The main disadvantage of octree-based compression methods is that the number of bits required increases dramatically with tree depth. This scheme is the basic scheme of point-cloud coding based on geometric information adopted by the MPEG G-PCC [1] standard group.

#### 2.1.2. Surface Approximation Based PCGC

Coding methods based on surface approximation are suitable for densely sampled and smooth surface point clouds. Compared with tree structure based coding methods, they can bring significant performance improvements at low bit rates. The point cloud itself is a sampling of the surface of the object, so the surface mesh model can be used to fit the distribution of the origin point cloud in space, and then the point cloud can be recovered by sampling on the surface. Surface approximation-based methods are often combined with octree-based space division. First, the point cloud is divided to obtain local point-cloud blocks, and then the plane is used to fit the local point cloud to achieve dimensionality reduction. In this way, only the edge corners of the plane need to be encoded, so it has higher encoding efficiency. This scheme is also one of the geometric coding schemes of MPEG G-PCC [1]. The disadvantage is that the plane-based coding method cannot achieve lossless coding due to the constant error of the plane approximation.

#### 2.1.3. Mapping-Based PCGC

Mapping-based encoding methods are mainly applied to dense dynamic point clouds. This type of method [15,16,17,18,19] maps the point cloud into a two-dimensional image, and then directly use a mature image and a video encoder for compression, which can achieve high encoding efficiency. The research mainly focuses on the mapping method, that is, how to realize the 3D to 2D mapping and its reconstruction as much as possible without loss; additionally, to help the mapped images develop a better spatial and temporal correlation in order to make better use of the efficient video coding methods and forecasting techniques. A more advanced mapping technique currently projects a patch of points with similar normal vectors onto the surface of a cube surrounding the point cloud, and then converts it into a depth map for compression. This patch-based projection avoids massive loss of occluded contiguous points. This scheme was adopted by the MPEG V-PCC [1] standard as a video-based point cloud coding standard.

### 2.2. Deep Learning Based PCGC

In recent years, with the rise of artificial intelligence technology, the methods using deep learning outperform them. In terms of point-cloud geometry compression, deep-learning-based approaches can be simply classified as voxel-based and point-based.

#### 2.2.1. Voxel-Based PCGC

This method extends the 2D Convolutional Neural Network (CNN) based image compression framework to 3D CNN-based volume model compression. Many such works [22,24] use 3D CNN to design codec networks. For the convolutional features, a learning-based entropy model is used for rate estimation. On the decoding side, the distortion is optimized by using a classification loss function with the reconstructed voxels being classified by a binary classification. There are also some studies, [26] representing volume models, based on Truncated Signed Distance Fields (TSDF), which can achieve a better geometry compression performance. Recently Wang et al. [23] proposed a multiscale geometric compression method, which achieves a good rate-distortion (R-D) tradeoff when compared to other approaches, while the distortion caused by quantization is ignored in the entropy coding stage. This will cause some feature information of the encoded point cloud to be lost, which leads to the reconstructed point cloud to ignore more details. Therefore, we further process the distortion through the proposed multi-layer residual module.

On the basis of voxelized point clouds, there are some works that use octree representation for PCC. For example, Huang et al. [27] and Que et al. [28] first divide the point cloud data into the octree structure, and the entropy model is constructed by exploiting the relationship between the nodes of the octree compression including ancestor nodes or neighbor nodes or their combination. Their works use 3D convolution to design a multilayer perceptron (MLP), which finally obtains the symbol prediction through the softmax layer.

#### 2.2.2. Point-Based PCGC

Voxel-based compression algorithms are limited by fixed precision constraints, which are difficult to be effectively applied to unevenly distributed and sparse point clouds, while neural networks that directly process point sets, such as PointNet have the ability to deal with them. Wen et al. [29] proposed a deep learning-based framework for lossy compression of geometric structures via hybrid representations of point clouds. The method firstly decomposes the input original point cloud into non-overlapping local sub-blocks adaptively through the decomposition and clustering of the adaptive octree. Then, a point cloud autoencoder network framework with quantization layers is proposed to learn compact latent feature representations from each sub-block. Subsequently, Zhu et al. [30] exploited regional similarity to achieve efficient lossy point cloud geometry compression. To this end, the input point cloud is divided into multiple local regions, and they are grouped into distinct clusters according to the region surface vectors, ensuring that the inter-cluster similarity is minimized and the intra-cluster similarity is maximized. Alignment transformation is performed on each cluster to predict non-reference regions of similar features from selected reference regions to achieve a considerable data reduction. In addition, Gao et al. [31] developed a more efficient point-based method for sparse point cloud compression, employing an end-to-end variational autoencoder structure to extract latent key points from point clouds using multi-scale neural graph sampling (NGS), taking neighboring structures as latent features. The decoder directly uses hierarchical convolution to gradually refine point reconstructions with aggregated features.

This kind of network [29,30,31] goes straight to taking the point cloud coordinates as an input and solves the disordered arrangement of point clouds through symmetric functions to learn latent features. The decoder directly generates the coordinates of the point cloud through a network such as a fully connected layer.

## 3. Method

**Figure 1:**
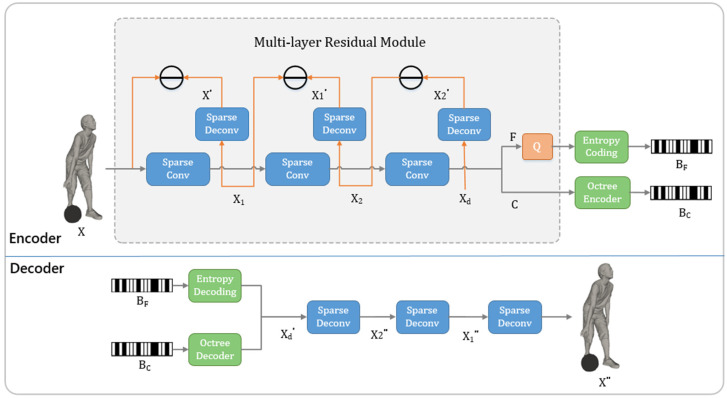
The architecture of the proposed method. Our multi-layer module is on the encoding side. The minus stands for geometric subtraction. C and F are the coordinate tensor and feature tensor, respectively. Q is quantization. B_C_ and B_F_ are the encoded bit-streams. Other symbols are described and explained in method.

The proposed multi-layer residual architecture is designed on the basis of the popular convolutional autoencoders via sparse convolution [25]. In order to reduce the loss of feature information caused by the quantization process, we introduced a multi-layer residual module at the encoder side to solve the distortion problem caused by the entropy encoding stage. We down-sample the input point clouds with the multi-layer residual module and reconstruct the points cloud in a hierarchical way. The holistic network structure is shown in Figure 1.

### 3.1. Sparse Convolution

We adopt a convolutional neural network based on sparse convolution [25] to exploit the sparsity of point clouds. Point cloud data are expressed as a set of sparse tensors, including their coordinates $C={\left\{\left(x_{i},y_{i},z_{i}\right)\right\}}_{i}$ and their associated features $F={\left\{f_{i}\right\}}_{i}$ in sparse convolution. Moreover, the convolution only extracts features of occupied coordinates. Its process is defined in [25] as follows:

(1) $$x_{t}^{\mathrm{out}}=\sum_{i\in K^{3}\left(t,C^{\mathrm{in}}\right)}W_{i}{x\;}_{t+i}^{\mathrm{in}}\;,\;\mathrm{for}\;t\in C^{\mathrm{out}}$$

where $C^{\mathrm{in}}\;\mathrm{and}\;C^{\mathrm{out}}$ are coordinates of input and output point clouds. $x_{t}^{\mathrm{in}}\;\mathrm{and}\;x_{t}^{\mathrm{out}}$ are feature vectors at coordinate u of input and output point clouds. $K^{3}\left(t,C^{\mathrm{in}}\right)=\{i|t+i\in C^{\mathrm{in}},i\in K^{3}\}$ is the definition of a 3D convolutional kernel centered on t with an offset of i. $W_{i}$ is the value of the convolution kernel at offset i.

**Figure 2:**
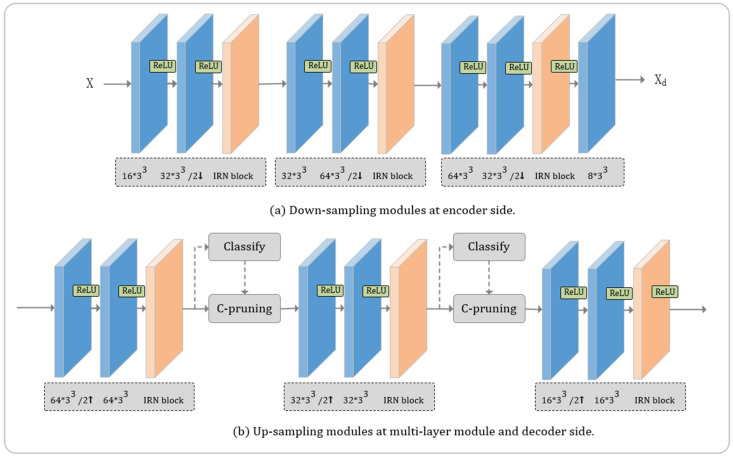
Sparse convolution based up-sampling and down-sampling modules. The upper picture is a down-sampling module based on sparse convolution, and the lower picture is an up-sampling module based on sparse convolution. “o × k^3^” represent a sparse convolutional layer with output channel o and convolution kernel 3 × 3 × 3. “2↑” and “2↓” represent up-sampling and down-sampling with a stride of 2, respectively.

Using sparse convolutions can help to reduce the complexity and efficiently extract features from point clouds. In this work, sparse convolution is utilized to efficiently down-sample to aggregate features and reconstruct the point clouds while up-sampling. Specifically, we utilize sparse convolutions on the encoder side for triple down-sampling and use in the up-sampling process of the multi-layer residual module as shown in Figure 2. In addition, in sparse convolution, we need to use geometric coding and octree coding for the down-sampled geometric coordinates C and feature information F, respectively.

### 3.2. Multi-Layer Residual Architecture

Encoder: At the encoder side of our whole point cloud compression architecture, the input point cloud X is down-sampled progressively, and the feature information F and coordinate information C of the X_d_ point cloud obtained are coded, respectively. Due to an additional quantization process in the entropy coding stage for F, this process makes our feature F compression lossy. Considering the distortion caused by quantization or the introduction of noise, we design a multi-layer residual architecture, as shown in Figure 1. A sparse conv module includes a sparse convolutional layer with stride 2 for down-sampling by a factor of 2, followed by an Inception ResNet block (IRN) [31] for a more efficient feature extraction. Each of the IRN blocks contains three consecutive IRN units. The above module reduces the amount of compressed data while extracting point cloud features.

For the multi-layer residual module, we perform specially up-sampling after each down-sampling. Specifically, the input point cloud X is first down-sampled to obtain $X_{1}$ by aggregating features, and then $X_{1}$ is up-sampled to obtain $X^{\prime }$ with voxel pruning, which imposes coarse-level constraints on the accuracy of the point cloud down-sampling process. Then geometrically subtract the geometric coordinates of X and $X^{\prime }$ to obtain the geometric residual. The process from $X_{1}$ to $X_{2}$ and from $X_{2}$ to $X_{d}$ which imposes finer level constraints on the accuracy of the point cloud down-sampling process is similar. For a given point cloud $M\left(x_{i},y_{i},z_{i}\right)\in X$, the corresponding point $M^{\prime }\left(x_{i}^{\prime },y_{i}^{\prime },z_{i}^{\prime }\right)$ is obtained after up-sampling, then their difference $\left(x_{i}^{\mathrm{res}},y_{i}^{\mathrm{res}},z_{i}^{\mathrm{res}}\right)$ is calculated by the geometric subtraction. We minimize the distortion of the difference to constrain the down-sampling accuracy. This way we obtain two bitstreams $B_{C}$ and $B_{F}$ through the encoder.

Decoder: At the decoder side, a sparse deconv module includes a sparse convolutional layer with stride 2 for up-sampling by a factor of 2, followed by an IRN [31] to better recover point cloud. Each of the IRN blocks contains three consecutive IRN units, as shown in Figure 2b. Then voxel pruning is performed according to the probability that it may be occupied. During the voxel pruning stage with layered reconstruction, we sort the resulting probabilities and consider the top k voxels as the most likely occupied voxels after a binary classification by a sparse convolution with output channel 1. We set k to be the number of ground truth labels to obtain the lowest distortion. We can obtain $X_{2}^{\prime \prime }$, $X_{1}^{\prime \prime }$ and $X^{\prime \prime }$ successively according to the decoded $X_{d}^{\prime \prime }$ in this way. In particular, the pruning of voxels, in the multi-layer residual module, does not depend on the probability different with the reconstruction when up-sampling.

### 3.3. Quantization and Entropy Coding Model

In this work, we use octree coding and entropy coding for the geometric information C and feature information F obtained by down-sampling, respectively. Before entropy coding, we need to quantize the feature information F. In order to ensure the differentiability of backpropagation, we add uniform noise:

(2) $$F^{\left(\eta \right)}=F+\eta ,{\;F}^{\left(\eta \right)}\sim \eta (F+\frac{1}{2},F-\frac{1}{2})$$

where η is random noise, $F^{\left(\eta \right)}$ and F are the original and quantized feature representations, respectively. $F^{\left(\eta \right)}$ follows a uniform distribution η centered on. For the entropy model stage, we use the probability density model to encode the quantized feature information, i.e., the full factorization model. Then under the hyperpriors [32,33] condition c, we use the Laplacian distribution L to approximate the probability density $P(F^{\left(\eta \right)}|c)$, which is defined as follows:

(3) $$P_{F^{\left(\eta \right)}|c}(F^{\left(\eta \right)}|c)=\underset{i}{\prod }c_{i}\left(L\left(\mu_{i},\sigma_{i}\right)\times \eta \right)$$

### 3.4. Joint Optimization Distortion

In our deep learning framework, we adopt joint optimization distortion for better compression performance and reconstruction quality. In detail, the loss of the multi-layer residual module is introduced while following the conventional rate-distortion. It is defined as follows:

(4) $$J_{\mathrm{loss}}=R+\lambda D+{\alpha L}_{\mathrm{res}}$$

where λ is used to control the bitrate. D measures the reconstruction loss using cross-entropy in Equation (5), and R is the bitrate of probability obtained by the prior model. $L_{\mathrm{res}}$ denotes the residual loss obtained by geometric subtraction at different precision levels and parameter α is the weight of $L_{\mathrm{res}}$ as in Equation (6):

(5) $$D=\frac{1}{M}\sum_{j}^{M}\frac{1}{N}\sum_{i}-(x_{i}\log\left(p_{i}\right)+\left(1-x_{i}\right)\log(1-p_{i}))$$

where M is the number of sampling layers, j is the sampling layer index. x indicates whether the voxel is occupied, occupied is 1 and empty is 0. p represents the probability that it may be occupied, p $\in \left(0,\;1\right)$ is activated by the sigmoid method:

(6) $$L_{\mathrm{res}}=\mathrm{sigmoid}\frac{1}{n}\sum_{i}^{n}{\left|\left|x_{i}-x_{i}^{\prime }\right|\right|}_{2}+{\left|\left|y_{i}-y_{i}^{\prime }\right|\right|}_{2}+{\left|\left|z_{i}-z_{i}^{\prime }\right|\right|}_{2}$$

where n is the number of point clouds in a batch. $\left(x_{i},y_{i},z_{i}\right)$ is the point cloud before down-sampling, and $\left(x_{i}^{\prime },y_{i}^{\prime },z_{i}^{\prime }\right)$ is the point cloud after up-sampling. We can obtain $\left(x_{i}^{\mathrm{res}},y_{i}^{\mathrm{res}},z_{i}^{\mathrm{res}}\right)$ by geometric subtraction and calculate the L2 norm sum. Therefore, $L_{\mathrm{res}}$ can be obtained by activating its mean through the sigmoid method.

## 4. Experiments

### 4.1. Experimental Setup

In training, we select 20,000 3D mesh models from ShapeNet [34] randomly. We generate points from the surfaces of the mesh. In addition, we adopt joint optimization distortion in Equation (4) and set the weight α for the residual loss to 10 for a better trade-off between the compression cost and the performance. To obtain models at different bit rates, we set λ from 0.15 to 5. We adjust the batch size to eight when training the model and optimize our proposed network with the help of the Adam [35] optimizer.

**Figure 3:**
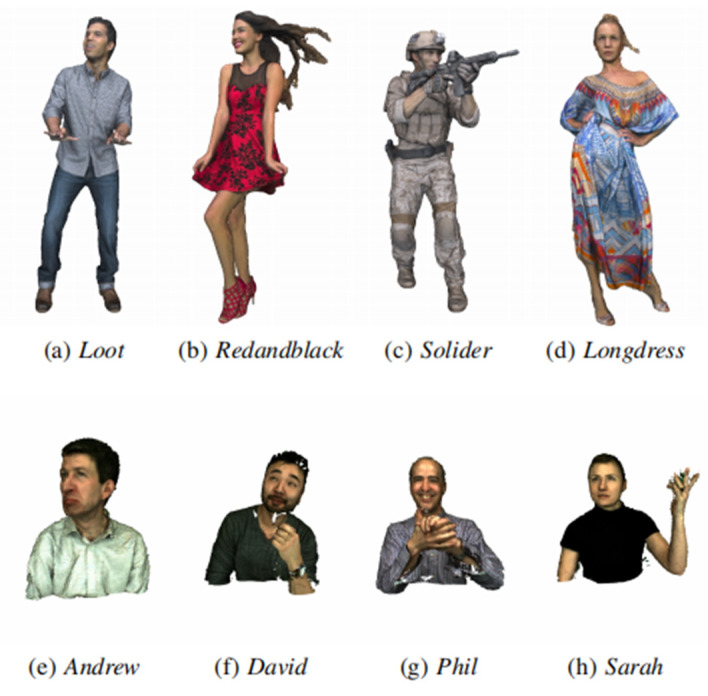
A preview of Testing Datasets.

In the tests, we use point cloud test data from 8i Voxelized Full Bodies (8iVFB) [36], Owlii dynamic human mesh and Microsoft Voxelized Upper Bodies (MVUB) [37]. These point cloud test data cover different types and scales, MPEG Common Test Condition(CTC) [38] and JPEG CTC [39] and use them in the compression work. The preview and details of the test point cloud are shown in Figure 3 and Table 1. Specifically, Class A (full bodies) exhibit a smooth surface and a complete shape, while Class B (upper bodies) present a noisy and incomplete surface (even having visible holes and missing parts).

### 4.2. Experiment Results

**Figure 4:**
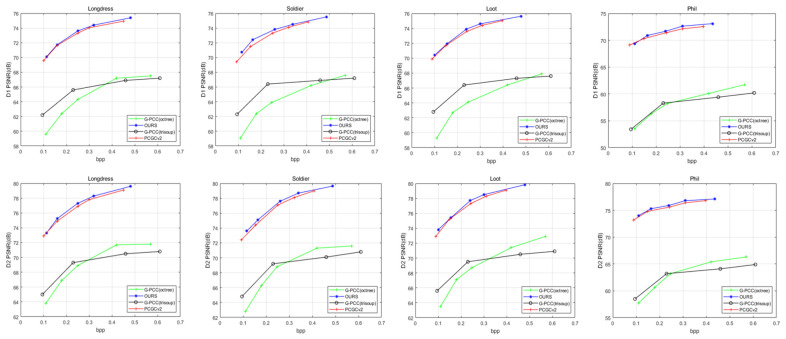
R-D performance including D1, D2 for G-PCC (octree), G-PCC (trisoup), PCGCv2 and our method.

Objective Quality Comparison: To validate the performance of our proposed method, we compared our multi-layer residual architecture with the state-of-the-art MPEG G-PCC [1] and V-PCC [1] schemes and followed the CTC [38] suggestions using TM13-v6.0 [40] to encode. In addition, we also compared our multi-layer residual architecture with the state-of-the-art PCGCv2 [20] work. Following the common objective quality measures, we use the bit rate as measurement which measures the bitsper input point (bpp). In our framework, the compression of geometric information occupies a small rate, while the feature information consumes most of the rate. Additionally, we adopt point-to-point distance (D1) [41,42] and point-to-plane distance (D2) as the distortion evaluation metrics. The rate-distortion curves including D1 and D2 are displayed in Figure 4. The BD-Rate and BD-PSNR gains are also displayed in Table 2 and Table 3, respectively.

As shown in Table 2 and Table 3, compared with point cloud compression schemes G-PCC (octree) [1] and G-PCC (trisoup) [1], our method successfully achieves on average more than 84% of BD-Rate gains and more than 72% of BD-Rate gains, respectively. Meanwhile, we obtain more than 38% of BD-Rate gains against V-PCC [1]. Moreover, compared to the state-of-the-art PCGCv2 [20], we achieve an average gain of about 10% in BD-Rate and show an improvement in terms of the performance on BD-PSNR.

**Figure 5:**
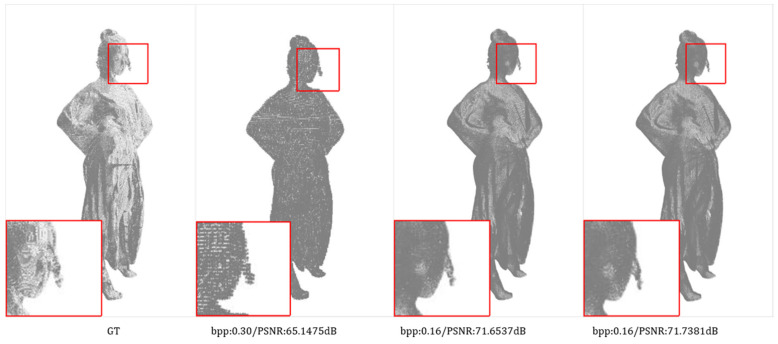

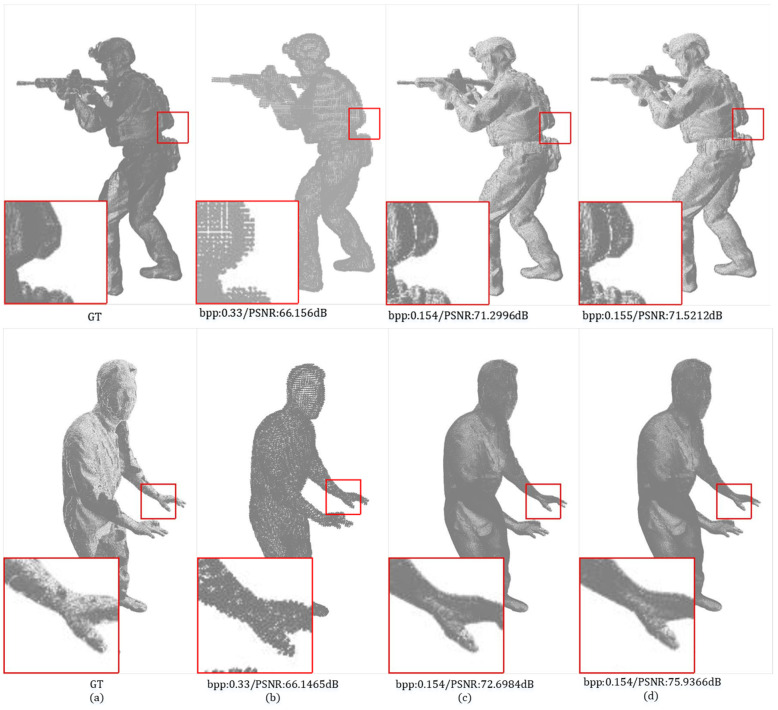
Subjective visualization of 8iVFB for comparison methods, our method and ground truth. (**a**) Ground Truth. (**b**) G-PCC (octree). (**c**) PCGCv2. (**d**) Ours.

**Figure 6:**
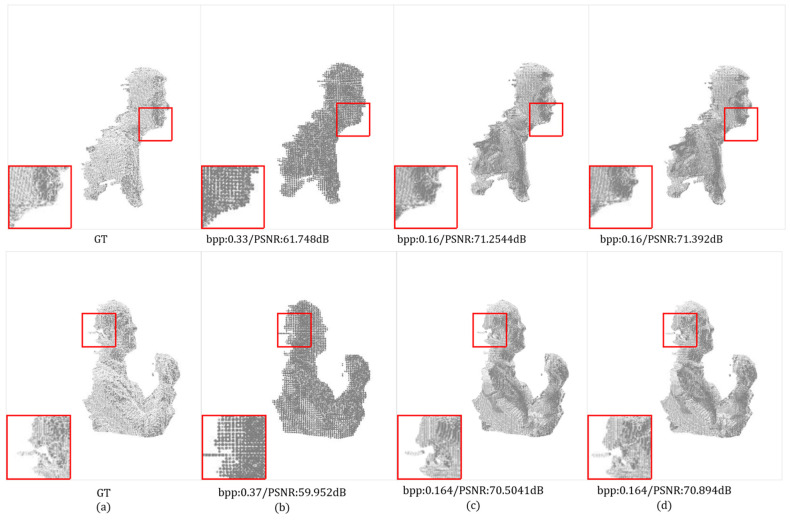
Subjective visualization of MVUB for comparison methods, our method and ground truth. (**a**) Ground Truth. (**b**) G-PCC (octree). (**c**) PCGCv2. (**d**) Ours.

Subjective Quality Comparison: We compare the subjective quality of reconstructed point clouds of the proposed method with G-PCC (octree) and the state-of-the-art method PCGCv2. We visualize the reconstructed point clouds of different methods when ours are at a lower bit rate or a similar bit rate, as shown in Figure 5 and Figure 6. It can be seen that we still have a higher reconstruction quality despite the low bit rate. Our reconstruction point cloud retains more detail and is smoother and more distributed in these details. For example, the hair part of Longdress, reconstructed by our method, is denser and finer in detail. Our method has reconstructed the contour of the backpack in the Soldier with clearer and more accurate edges. In addition, the jaw of Andrew, reconstructed by our method, is much finer and smoother.

### 4.3. Ablation Study

Weight of multi-layer residual distortion: In the training process, in order to make the model have a better compression performance, we set different weights for the multi-layer residual loss. To estimate the effect of multi-layer residual loss on model performance, we set the parameter α as α = 5, α = 10, α = 15 and α = 20. Experiments show that when the parameter α = 10, the quality of the reconstructed point cloud of the model is the best, as shown in Table 4 and Table 5. Therefore, while training, we set the parameter α to 10 to obtain the best R-D tradeoff.

### 4.4. Complexity Discussion

In terms of complexity, our experiments are conducted on a workstation equipped with Intel Core i7-9700K CPU and an Nvidia GeForce GTX 1080 GPU. Our prototype, on average, takes 1.59 s for encoding and 5.44 s for decoding on an 8iVFB dataset at the highest bitrate, while the encoding and decoding time for G-PCC (octree) is about 1.6 s and 0.6 s, respectively, as shown in Table 6. The reason for this is that the point cloud reconstruction structure based on a binary classification needs to deal with more voxels. Additionally, we need to point out that our method is implemented in a prototype, which can be further optimized and improved in our future work. The number of parameters of our model is about 894 kb, while the parameter size of PCGCv2 is 778 kb, which shows our method has a relatively lightweight network when compared to other popular algorithms.

## 5. Conclusions

In this paper, we proposed a multi-layer residual module for point-cloud geometry compression, which imposes different level constraints on the accuracy of the point cloud sampling process to deal with the distortion caused by quantization or the introduction of noise. We take advantage of the sparsity of point clouds, with sparse convolutions for the complexity reduction in the space and time. For point cloud applications, such as autonomous driving, a mainstream 64-line LiDAR can produce 1 TB raw point cloud data, which brings a great burden to its transmission and processing. Additionally, our method can greatly reduce it to about 1/130 of its original size. Experimental results validate that our method improves the reconstruction quality of point clouds and preserves more detail. Meanwhile, our method outperforms MPEG G-PCC and V-PCC substantially at a BD-Rate gain. Additionally, compared to the state-of-the-art PCGCv2, our method can achieve an average gain of about 13% and 10% in BD-Rate with D1 and D2 distortion criteria. In our future work, we can further improve the entropy coding module by constructing a more accurate context probabilistic model utilizing 3D neighboring points. One future research direction is to generalize our multi-layer residual architecture to other point cloud processing tasks, for example, point cloud denoising, point cloud segmentation and classification.

## Figures and Tables

**Table 1:** Details of Testing Datasets.

Point Cloud		Points	Precision	Frame
Class A	Longdress	857,966	10	1300
	Soldier	1,089,091	10	690
	Loot	805,285	10	1200
	RedandBlack	757,691	10	1550
Class B	Andrew	279,664	9	1
	Phil	370,798	9	1
	David	330,791	9	1
	Sarah	302,437	9	1

**Table 2:** BD-Rate gains of some test data against G-PCC (octree), G-PCC (trisoup), V-PCC and PCGCv2 using D1 and D2 distortion measurement.

Point Cloud	D1 (p2point)				D2 (p2plane)			
	G-PCC (Octree)	G-PCC (Trisoup)	V-PCC	PCGCv2	G-PCC (Octree)	G-PCC (Trisoup)	V-PCC	PCGCv2
Longdress	−91.35%	−77.30%	−39.93%	−8.65%	−84.76%	−73.37%	−42.06%	−6.96%
Soldier	−90.37%	−76.88%	−39.50%	−13.25%	−84.74%	−72.92%	−41.97%	−10.04%
Loot	−91.22%	−81.65%	−39.67%	−13.34%	−85.19%	−73.44%	−42.13%	−10.23%
RedandBlack	−90.36%	−81.21%	−39.61%	−12.75%	−85.03%	−73.28%	−42.04%	−9.37%
Andrew	−92.14%	−87.38%	−60.94%	−14.80%	−83.17%	−87.79%	−53.61%	−11.30%
Phil	−92.35%	−87.97%	−61.06%	−14.32%	−83.79%	−80.06%	−53.88%	−10.25%
David	−92.41%	−86.73%	−61.55%	−13.79%	−82.93%	−82.45%	−54.17%	−10.17%
Sarah	−93.16%	−86.66%	−60.39%	−14.64%	−83.61%	−86.36%	−53.65%	−11.40%
Average	−91.67%	−83.22%	−50.33%	−13.19%	−84.40%	−76.71%	−45.94%	−9.97%

**Table 3:** BD-PSNR gains using D1 distortion of some test data against G-PCC (octree), G-PCC (trisoup), V-PCC and PCGCv2.

Point Cloud	BD-PSNR			
	G-PCC (Octree)	G-PCC (Trisoup)	V-PCC	PCGCv2
Longdress	8.89	7.91	3.11	0.24
Soldier	9.29	7.43	3.59	0.41
Loot	9.66	7.31	3.52	0.40
RedandBlack	8.41	6.94	3.22	0.35
Andrew	9.74	11.15	3.95	0.33
Phil	10.43	12.10	4.36	0.51
David	9.54	10.57	3.14	0.40
Sarah	8.94	9.78	3.36	0.37
Average	9.36	9.13	3.53	0.38

**Table 4:** BD-Rate gains with different weights of multi-layer residual module.

Point Cloud	BD-Rate			
	α=5	α=10	α=15	α
Longdress	−4.32%	−6.96%	−6.76%	−4.55%
Soldier	−7.41%	−10.04%	−8.69%	−7.28%
Loot	−9.56%	−10.23%	−9.15%	−10.13%
RedandBlack	−5.51%	−9.37%	−7.28%	−7.73%
Average	−6.70%	−9.15%	−7.94%	−7.42%

**Table 5:** BD-PSNR gains with different weights of multi-layer residual module.

Point Cloud	BD-PSNR			
	α	α=10	α=15	α=20
Longdress	0.15	0.24	0.21	0.19
Soldier	0.23	0.41	0.37	0.36
Loot	0.28	0.40	0.32	0.33
RedandBlack	0.21	0.36	0.30	0.31
Average	0.22	0.35	0.30	0.28

**Table 6:** Average running time (s) of different methods.

	G-PCC (Octree)	G-PCC (Trisoup)	V-PCC	PCGCv2	Ours
Encoding	1.60	8.16	103.41	1.56	1.59
Decoding	0.60	6.58	0.67	5.42	5.44

## Data Availability

Not applicable.
